# Electrogene therapy with interleukin-12 in canine mast cell tumors

**DOI:** 10.2478/v10019-010-0041-9

**Published:** 2010-09-22

**Authors:** Darja Pavlin, Maja Cemazar, Andrej Cör, Gregor Sersa, Azra Pogacnik, Natasa Tozon

**Affiliations:** 1 University of Ljubljana, Veterinary Faculty Ljubljana, Ljubljana, Slovenia; 2 Institute of Oncology Ljubljana, Department of Experimental Oncology, Ljubljana, Slovenia; 3 University of Primorska, College of Health Care Izola, Izola, Slovenia

**Keywords:** electroporation, electrotransfection, electrogene therapy, mast cell tumors, IL-12, IFN-γ

## Abstract

**Background:**

Mast cell tumors (MCT) are the most common malignant cutaneous tumors in dogs with extremely variable biological behaviour. Different treatment approaches can be used in canine cutaneous MCT, with surgical excision being the treatment of choice. In this study, electrogene therapy (EGT) as a new therapeutic approach to canine MCTs, was established.

**Materials and methods.:**

Eight dogs with a total of eleven cutaneous MCTs were treated with intratumoral EGT using DNA plasmid encoding human interleukin-12 (IL-12). The local response to the therapy was evaluated by repeated measurements of tumor size and histological examination of treated tumors. A possible systemic response was assessed by determination of IL-12 and interferon- γ (IFN-γ) in patients’ sera. The occurence of side effects was monitored with weekly clinical examinations of treated animals and by performing basic bloodwork, consisting of the complete bloodcount and determination of selected biochemistry parameters.

**Results:**

Intratumoral EGT with *IL-12* elicits significant reduction of treated tumors’ size, ranging from 13% to 83% (median 50%) of the initial tumor volume. Additionally, a change in the histological structure of treated nodules was seen. There was a reduction in number of malignant mast cells and inflammatory cell infiltration of treated tumors. Systemic release of IL-12 in four patients was detected, without any noticeable local or systemic side effects.

**Conclusions:**

These data suggest that intratumoral EGT with plasmid encoding IL-12 may be useful in the treatment of canine MCTs, exerting a local antitumor effect.

## Introduction

Mast cell tumors (MCT) are the most common malignant cutaneous tumors in dogs, accounting for around 21% of all cutaneous tumors.^1^ Cutaneous MCT have extremely variable biological behavior, from low-grade tumors to highly invasive lesions with high metastatic potential, which makes proper staging and treatment of MCT very challenging.

Treatment options for canine MCT depend on prognostic factors, primarily the histological grade of the tumor and clinical stage of the disease. The treatment of choice for MCT is wide surgical excision, when possible, which results in excellent prognosis for well-differentiated MCT.^1^ Poorly differentiated or anaplastic MCT carry a poor prognosis and in these tumors aggressive surgical treatment should be followed by other treatment modalities, *e.g*. radio- or chemotherapy.^1^ In dogs, where it is not possible to perform surgical excision and in cases with advanced stages of disease, systemic chemotherapy is the most appropriate treatment option.^1^,^2^

One of the newer therapeutic approaches for local tumor control is electrochemotherapy (ECT), which has already been established as a successful treatment option for different histological types of canine tumors, including MCT.^3^ It employs intralesional or systemic injection of the chemotherapeutic agent bleomycin or cisplatin, followed by local delivery of electric pulses to the tumor nodule, which significantly increase uptake and cytotoxicity of chemotherapeutic drugs.^4^–^6^ The procedure is based on electroporation of the cell membrane, achieving a transient increase in its permeability, thus allowing intracellular uptake of chemotherapeutic drugs from the extracellular space.^7^

The same principle can also be used for intracellular delivery of other molecules, for example plasmid DNA. Combining direct injection of plasmid DNA containing a therapeutic gene into target tissue, together with local delivery of electric pulses is called electrogene therapy (EGT).^8^–^10^ In veterinary medicine, it has already been used for delivery of different transgenes into skeletal muscle^11^–^13^ and intratumorally.^14^,^15^

IL-12 exhibits a range of biological activities, potentially important in immunotherapy of cancer. These include, for example, activation of natural killer cells, induction of IFN-γ, inhibition of angiogenesis and stimulation of nitric oxide production.^16^ Gene therapy using *IL-12* has already shown remarkable antitumor activity in different tumor models at the preclinical level, and has already progressed to a number of clinical trials in both human and veterinary medicine. 17–21

The aim of our study was to evaluate the local antitumor effect, systemic transgene release and possible side effects of EGT with the therapeutic plasmid encoding human IL-12 in canine MCT. For this purpose, a plasmid encoding human IL-12 was injected intratumorally into spontaneously occurring superficial nodules of MCT in 8 patients, followed by application of electric pulses. Local response to therapy was evaluated by regular measurements of tumor size and histological assessment of excised tumor nodules. Systemic transgene release was determined by measurements of IL-12 and IFN-γ in patients’ sera. Possible side effects of the procedure were monitored by regular determination of selected hematology and biochemistry parameters.

## Materials and methods

### Animals

All animals in this study were referred to the Veterinary Faculty of Ljubljana in February and March 2006 for evaluation of cutaneous or subcutaneous tumor nodules. Eight patients that corresponded to inclusion criteria for the clinical study were included. The study cohort was comprised of 3 intact males and 5 spayed females of 6 different breeds (3 German boxers, and one of each: cross-breed, toy poodle, French bulldog, Siberian husky and bullterrier), their age ranging from 5 to 16 years (Table 1). Inclusion criteria for the study comprised at least one cytologically or histologically confirmed MCT, good general health status of the animal with the basic hematology and biochemistry profile within reference limits and normal renal and cardiovascular function. Animals included in the study were either ones which were planned for surgical excision of the tumor nodule as a part of standard therapeutic procedure and their owners agreed to the EGT procedure prior to surgery, or had recurrent disease in which other conventional therapy methods were already exhausted by previous treatments, or their owners refused any other type of standard treatment at the time of inclusion. Prior to inclusion, written consent for participation in the clinical study for each animal was obtained from their owners. The study was approved by the Ethical Committee at the Ministry of Agriculture, Forestry and Food of the Republic of Slovenia (approval No. 323-451/2004-9).

Clinical examination of each animal was performed before the treatment. Fine needle aspiration biopsy of tumor nodules, as well as of local lymph nodes, was taken and cytological examination of samples was performed. In all animals, staging was performed according to modified WHO staging criteria^22^ with examination of thoracic radiographs, abdominal ultrasonography and basic bloodwork. Basic bloodwork consisted of a complete blood count with differential white blood cell count, which was performed using an automated laser hematology analyzer (Technicon H^*^1, Bayer, Germany) with species-specific software (H^*^1 Multi-Species V30 Software). The automated chemistry analyzer Technicon RA-XT (Bayer, Germany) was used for determination of the following biochemical parameters: blood urea nitrogen (BUN), creatinine, serum alkaline phosphatase (SAP) and alanine aminotransferase (ALT). Additionally, serum concentrations of human IL-12 and canine IFN-γ were determined using ELISA kits (Human IL-12 Quantikine ELISA Kit and Canine IFN-γ Quantikine ELISA Kit, respectively, both R&D Systems, Minneapolis, MN, USA).

A total of 11 tumor nodules were treated with EGT in the 8 patients which were included in the study. Two dogs received only one EGT, four dogs received 2 EGTs, with the second EGT session delivered one week after the first treatment, and one dog received 3 EGT sessions, each one week apart. In the remaining dog, four sessions were performed, each one month apart (Table 2).

In the patients, EGT was performed either as a single therapy (2/8 patients) or it was followed with one of the following therapies: standard surgical removal of MCT nodules (4/8 patients), chemotherapy (2/8 patients) or ECT (one nodule in a patient with two MCTs) (Table 2). Treated tumors were surgically removed one week after the last EGT session. In patients with systemic chemotherapy, 3 and 4 cycles of CCNU (lomustine, 60–90 mg/m^2^, every 3 weeks) were delivered, starting two weeks after the last EGT session. ECT was performed one week after the last EGT session with intratumoral application of cisplatin (Cisplatyl, Aventis, Paris, France) at a dose 1 mg/cm^3^ of tumor tissue, followed by application of electric pulses (8 pulses of 100 μs duration at an amplitude of 1300 V/cm and frequency 1 Hz), using the same electric pulse generator as described below.

In animals in which histological samples of tumors were obtained either as part of the diagnostic procedure or with surgical excision after EGT, tumor samples were histologically evaluated. Histological grading was established using Patnaik’s histological criteria.^22^

### Plasmid preparation

The pORF-hIL-12 plasmid (InvivoGen, Toulouse, France), encoding human IL-12, was selected according to available data indicating that canine and human IL-12 share approximately 90% genetic identity based on amino acid sequence analysis.^23^ It has already been shown that human IL-12 activates proliferation of canine peripheral blood mononuclear cells (PBMC) in an *in vitro* setting and triggers a number of immune responses in canine PBMC.^24^ The plasmid was prepared using the Qiagen Endo-Free kit (Qiagen, Hilden, Germany), according to the manufacturer’s instructions and diluted to a concentration of 1 mg/ml. Purified plasmid DNA was subjected to quality control and quantity determinations, performed by agarose gel electrophoresis and by means of spectrophotometry.

### Electrogene therapy procedure

EGT was performed in animals under general anesthesia, which was induced with propofol (Propoven 10 mg/ml, Fresenius Kabi Austria GmbH, Graz, Austria) and maintained with isoflurane (Forane, Abbott Laboratories LTD, Queensborough, United Kingdom). During the anesthesia, animals received Harmann’s solution (B. Braun Melsungen AG, Melsungen, Germany) at a rate of 10 ml/kg of body weight/h.

In animals under general anesthesia, hair overlying tumor nodules was removed, carefully avoiding any unnecessary manipulation of tumors which could lead to degranulation of mast cells. Each nodule was measured in three perpendicular directions (a, b, c) and the tumor volume was calculated using the formula: V = a x b x c x π/6. A sterile solution of plasmid was injected into the tumor with a 1 ml syringe and 22 G needle. The dose of intratumorally injected plasmid ranged from 0.5 to 1 mg/cm^3^ of tumor tissue per one EGT session for tumors with volumes ranging from 0.1 cm^3^ to 2.5 cm^3^. In smaller tumors (< 0.1 cm^3^) and in larger tumors (> 2.5 cm^3^), an arbitrary dose per tumor nodule was set, being 0.1 mg for smaller and 1 mg for larger tumors (Table 2). Ten minutes after plasmid injection, electric pulses were delivered using the electric pulse generator Cliniporator^™^ (IGEA s.r.l., Carpi, Italy), using needle electrodes (2 arrays each composed of 4 electrodes with a 4-mm distance between them). One high voltage pulse was delivered (1 x 1200 V/cm, 100 μs), immediately followed by 8 low voltage pulses (8 x 50 ms, 140 V/cm, 2 Hz). After the electrogene procedure, all dogs received single intravenous application of carprofene (Rimadyl, Pfizer Animal Health, Dundee, United Kingdom; 4 mg/kg of bodyweight). When they fully recovered from anesthesia, they were released from hospital. Prior to release into the home environment, animals received Elizabethan collars in order to prevent any wound licking. Furthermore, treated tumor nodules were protected with suitable dressing to prevent any possible contact of humans or animals with the electroporated area.

### Evaluation of response to therapy and possible side effects

Animals were examined one, two and four weeks after each EGT session and thereafter monthly. At each examination, a local as well as systemic response to the therapy was determined, along with observation for possible side effects.

The local response to therapy was evaluated with repeated measurements of tumor size as described above and calculation of tumor volumes. Additionally, in animals which underwent surgical excision of tumors, histological examination of tumor samples was performed. The systemic response to the treatment was assessed by determination of IL-12 and IFN-γ in patients’ sera.

The possible occurrence of local or systemic side effects was evaluated at each follow- up with clinical examination of the animals and careful assessment of the appearance of the electroporated area for any possible clinical signs, including erythema, oedema, pain, secretions, necrosis, etc. Furthermore, blood samples were taken for the same bloodwork as before the procedure.

### Statistical analysis

Statistical analysis was performed using SigmaPlot software (Systat Software, Inc., Richmond CA, USA). To determine the significance of differences in tumor volumes of MCT before and after the treatment, a Friedman repeated Measures Analysis of Variance on Ranks was performed. Values of P<0.05 were considered significant.

## Results

### Response to the therapy

Before EGT, tumor volumes ranged from 0.03 to 25.4 cm^3^. Treated nodules reached the smallest volume one to two weeks after the last EGT procedure (Table 2), with their volumes ranging from 0.005 to 18 cm^3^, which was statistically significantly smaller compared to the volumes before EGT. One week after the last EGT session and before induction of any other therapeutic procedure, the tumor volume was reduced in 9/11 treated tumors, it had not changed in 1/11 treated tumors and progressed in 1/11 treated tumors. In nodules where reduction of tumor volume was achieved, it ranged from 13% and up to 83% of the initial value (median 50%).

In two patients (#1 and #6) with a total of three tumor nodules, EGT with *IL-12* was not followed by any other treatment (Table 2). In both patients, the tumor nodules reduced in size and treated patients responded to therapy with stable disease throughout the very long observation period (36 and 44 months).

Two patients (#3 and #8) received systemic chemotherapy with CCNU (Table 2). In one of these patients (#3) with stage II disease (regional lymph node involvement), stable disease with reduction in tumor volume by 50% was achieved with regression of detectable mast cell infiltration of lymph nodes and without any signs of distant metastases throughout the 12-month observation period. The second patient, (#8), treated with a combination of EGT and systemic chemotherapy had recurrent stage III disease, unresponsive to any treatment and was euthanized 5 months after EGT due to progress of the disease with systemic clinical signs.

In one patient (#4) with two tumor nodules, EGT in one nodule was followed by surgical removal of the tumor and in the other, due to its location in the perineum, EGT was followed by ECT, achieving a complete response in the treated nodule (Table 2).

In four patients (#2, #4, #5 and #7), surgical removal of 4 grade II and one grade III tumor nodules was performed one week post-EGT (Table 2). After surgery, three had a complete response to therapy without any signs of local recurrence or metastatic disease in over a 1-year observation period. The remaining one (#7), with stage III disease, had progression of clinical signs and was euthanized 2 months after EGT.

### Histology of the tumors

All surgically removed tumors underwent histological examination. The control group represented MCT samples taken with a biopsy from the same tumor nodule during the diagnostic workup before inclusion into the clinical study or from untreated tumor nodules which were simultaneously removed in patients with multiple nodules.

**FIGURE 1.**
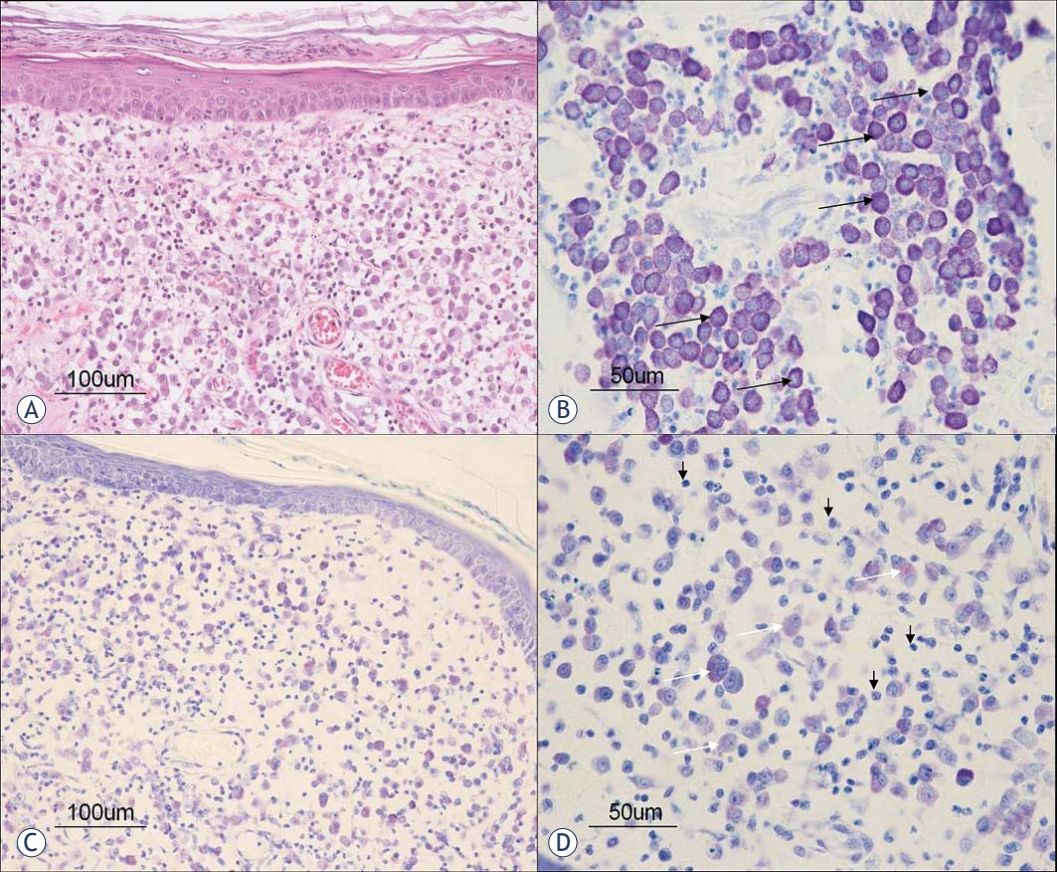
Histological pictures of MCTs before (A and B) and after (C and D) EGT. A. Tumor mast cells are loosely arranged in the dermis without epidermal invasion (haematoxylin and eosin staining). B. Tumor mast cells (arrow) have a well granulated metachromatic cytoplasm (toluidine blue staining). C. Decreased number of mast cells in the dermis after the treatement (toluidine blue staining). D. Note the degranulated tumor mast cells with metachromatically weakly stained cytoplasm (white arrows) intermingled with numerous inflammatory cells (black arrows) (toluidine blue staining).

Histological analysis of MCT prior to *IL-12* EGT showed nonencapsulated dermal and/or subcutaneous infiltrative growing masses composed of round cells arranged in densely packed cords. Most malignant mast cells were recognized in H&E stained slides by their cytoplasmic light gray-blue granules. Granules stained metachromatically with cationic dyes (toluidine blue staining). Most cells had a single nucleus, however same binucleated mast cells were also found. Among mast cell cords, variable numbers of diffusely distributed or aggregated eosinophils were seen. After the treatment, the distributions of viable malignant mast cells were reduced in comparison to the pre-treatment samples (Figure 1A, B). Instead of mast cells in the dermis and subcutis, clusters of leukocyte infiltration were found. Large areas of mostly lymphocytes and plasma cells with eosinophilic cytoplasm and perinuclear halos were seen (Figure 1C, D). No similar infiltrates were found in samples of untreated lesions. Among immune cells, some degranulated mast cells were noticed without prominent neutrophils or eosinophils.

### Hematology and systemic release of cytokines

In order to evaluate any possible systemic effects of the therapy, serum concentrations of human IL-12 and canine IFN-γ were measured at regular time-points after the procedure.

IL-12 was detected in 5 samples from 3 patients, with serum concentrations ranging from 1 to 12.2 pg/ml. IFN-γ was detected in 5 samples from 2 patients, with concentrations ranging from 123.0 to 815.6 pg/ml. IL-12 and/or IFN-γ were detected in a total of 4 patients. All positive samples were collected after the EGT procedure; in samples, taken before the procedure, neither of the cytokines was detected. The highest systemic concentrations of transgene products were detected in patient #3. In this dog, all 3 post-EGT samples were positive for both IL-12 (2.3 pg/ml to 12.2 pg/ml) and IFN-γ (170 pg/ml to 388.1 pg/ml) (Table 3).

In order to evaluate possible side effects of the procedure, clinical examinations of patients were carried out on a regular basis, with careful examination of the electroporated area and selected bloodwork.

We did not detect any evident side effects, either locally or systemically. All patients, which responded to treatment remained in good clinical condition throughout the observation period, without any additional clinical signs of disease. All analyzed blood parameters remained within the normal reference range. The few alterations in bloodwork parameters which occurred were only minimal and clinically irrelevant (for example, mild haemoconcentration in two samples). In two patients, slight elevations of SAP and/or ALT were detected in samples, obtained 1 and 2 months after the procedure, but they were considered side effects of CCNU chemotherapy, which was started 2 weeks after EGT. One of these two patients was euthanized due to progressive disease, unresponsive to all therapies whilst in the other, serum concentrations of both SAP and ALT returned within the reference range after discontinuation of chemotherapy. Details of hematology and the biochemistry profile in patient # 3, in which the best systemic response to treatment with the highest number of serum samples positive to IL-12 and IFN-γ was achieved, are summarized in Table 3.

## Discussion

Our study demonstrates that intratumoral EGT with *IL-12* in canine MCT elicits good local anti-tumor effects in treated animals without any noticeable side effects. Local antitumor effects of this therapy can be seen as significant reduction in tumor size (median reduction of the pretreatment tumor volumes was 50%) and change in histological structure with reduction in the number of malignant mast cells coupled with infiltration of inflammatory cells in treated tumors. We also demonstrated that systemic release of the transgene product is possible after intratumoral EGT.

EGT is a novel treatment in medicine which has already entered the clinical stage of research in human oncology^20^, and is also gaining some interest in veterinary medicine.^11^–^15^,^25^ The results of EGT with various therapeutic genes including plasmid encoding IL-12 in the treatment of tumors are promising.^21^ In a recent human clinical study, EGT with *IL-12* plasmid in the treatment of melanoma patients showed local as well as systemic antitumor effects with regression of tumor nodules and with minimal systemic toxicity.^20^

In our study, we treated spontaneously occurring cutaneous nodules of MCT in eight canine patients utilizing locally applied EGT with a therapeutic gene encoding human IL-12. The majority of treated nodules regressed in size after the EGT procedure by 50% (median) around 1–2 weeks after the last EGT session. These results can be compared to published results by Rakhmilevich *et al.* on growth delay of murine P815 mastocytoma^26^ after bioballistic *IL-12* gene therapy and Heinzerling *et al.*^17^, who utilized a similar approach for treatment of melanoma in horses with direct intratumoral application of plasmid DNA encoding human IL-12 without subsequent electroporation. In the murine mastocytoma model, a 60% reduction in volume was achieved three weeks after therapy. In equine melanomas, the mean reduction in size was 69% of the initial volume. It is possible that in dogs more repetitions of treatment sessions would result in even more pronounced tumor regression, since in the murine MCT model 6 repetitions whilst in horses up to 3 repetitions of treatment were necessary to reach a significant reduction in tumor size.

Histological analysis revealed a noticeable change in tumor morphology after EGT with *IL-12*. Beside reduction in the number of malignant mast cells, the most prominent feature of treated tumors was diffuse infiltration of tumor tissue with lymphocytes and plasma cells as well as degranulation of remaining mast cells. These histological changes are in accordance with other studies employing intratumoral *IL-12* gene therapy with different delivery systems, where intra- and peritumoral lymphocytic infiltration was found to be a major contribution to histological changes in treated nodules.^17^,^20^,^27^ The importance of lymphocytic infiltration of treated tumors after intratumoral *IL-12* EGT was shown in a variety of preclinical studies.^27^,^28^ It was established that this mode of therapy does not elicit any antitumor effect in athymic mice deficient in T cells in contrast to immunocompetent mice, indicating the vital role of T lymphocytes in the antitumor activity of local *IL-1*2 EGT.^27^,^28^ In our study, intratumoral *IL-12* EGT resulted in an immunological response with lymphocytic infiltration of treated tumors, which can be further indication that plasmid encoding human IL-12 is biologically active in dogs *in vivo*, as it was proposed to be in *in vitro* settings.^24^

The importance of the systemic action of IL-12 after local delivery in addition to local intratumoral accumulation of IL-12 has already been shown, demonstrating that circulating IL-12 is responsible for systemic antitumor effects, *e.g*. an antitumor effect on distant untreated tumors and prevention of distant metastases.^29^–^32^ Therefore, systemic release of the transgene product would be extremely favorable in clinical settings, expanding local antitumor therapy into systemic treatment. In our study, systemic release of human IL-12 was detected in only three patients. Even though at the preclinical level there is contradictory data on the possibility of systemic transgene release after intratumoral *IL-12* EGT, in two studies on induced transmissible veneral tumors in dogs^14^,^15^, similar concentrations of human IL-12 as in our three patients were detected. Therefore, further investigations are warranted to determine conditions for achieving systemic effects of intratumoral EGT with *IL-12* in dogs, since some release of IL-12 from treated tumors is clearly possible.

In treated patients we paid attention to two possible groups of adverse side effects. *IL-12*-based intratumoral EGT could lead to degranulation of mast cells, causing histamine release from granules, which is one of the major concerns of any mechanical manipulation of MCT. It can result in either local side effects, demonstrated as peritumoral swelling, edema and erythema, or in systemic clinical signs, for example, gastrointestinal ulceration, or even life-threatening hypotension, arrhythmias and bronchospasm. We did not observe any of these side effects, even though moderate mechanical manipulation of tumors could not be avoided since penetrating needle electrodes, which had to be inserted intratumorally, were used for EGT.

The second group of possible side effects is connected to systemic IL-12 toxicity. It has been shown that systemic administration of recombinant protein IL-12 is associated with multiple serious adverse side effects, including renal and systemic toxicity. High-dose levels were also linked to temporary immune suppression, which would not be favorable for effective immunotherapy.^33^ Local intratumoral *IL-12* EGT was associated with significantly less adverse effects, while exhibiting a pronounced antitumor effect, as demonstrated by Heller *et al.* on a mouse melanoma model.^34^ Even so, monitoring renal function with selected laboratory parameters (*e.g*. serum concentrations of BUN, creatinine) was recommended in any IL-12-based therapy.^34^ In our study, all monitored hematological and biochemical parameters in blood samples remained within reference limits throughout the whole observation period with only few transient clinically nonsignificant and nonspecific alterations, which could be attributed to other factors. The clinical status of all animals that responded to therapy remained unaltered and they didn’t show any changes in appetite, water intake and general behavior.

In conclusion, the results of our study demonstrate that intratumoral *IL-12* EGT in canine MCT is a feasible, simple and safe therapeutic procedure, which exerts local transgene expression with systemic release of the encoded protein, making it a promising treatment for canine patients with MCT. However, further refinement for effective use of this method in treatment of MCT is needed, with emphasis on optimization of the treatment protocol, including determination of appropriate dosage of the plasmid used, as well as the best possible number of EGT repetitions and optimal time interval between them.

## Figures and Tables

**Table 1.** Summary of dogs’ characteristics and histories

**Pt. No.**	**Breed**	**Age (yrs)/Gender**	**Body weight (kg)**	**Duration of clin. signs (months)**	**Tumor location**	**Clinical stage**	**Cytology**	**Histology**	**Previous treatment**	**Response to previous treatment**	**No. of tumor nodules**
1	Toy poodle	16/FS	6	3	back	I	MCT	N/A	no		1
2	Boxer	7/M	42	4	scapula	I	MCT	N/A	no		1
3	Cross-breed	10/FS	16.5	12	hind leg	II	MCT	N/A	no		1
4	Boxer	10/FS	27	6	perineum, back	II	MCT	grade II	no		2
5	Boxer	10/M	35	2	scrotum, scapula	III	MCT	grade II	no		4
6	Bullterrier	5/FS	20	>12	hind leg	II	MCT	grade II	surgery 2x chemotherapy (vincristine, CCNU)	recurrence	2
7	Siberian husky	11/FS	18	< 1	fore leg	III	MCT	grade III	no		1
8	French bull-dog	7/M	12	4	multicentric	III	MCT	grade III	surgery chemotherapy (vincristine)	recurrence PD	>10

FS, spayed female; MCT, mast cell tumor; M, male; CCNU, lomustine; PD, progressive disease

**Table 2.** Details of EGT treatment and response to therapy

**Pt No.**	**Nodule**	**Tumor volume before EGT (cm^3^)**	**Tumor volume after EGT^1^** **(cm^3^)**	**No. of sessions**	**Dose of pIL12 per EGT session (mg)**	**Dose of plasmid per body weight (mg/kg)^2^**	**Post EGT therapy**	**Follow up after 1^st^ EGT (months)**	**Response at the end of follow up**	
1	1	0.25	0.1	2	0.15/0.15	0.05	/	36	SD	Euth., not related to MCT
2	1	2.3	2.0	1	0.5	0.01	Surgery	24	CR	without recurrence
3	1	3.2	1.6	2	1.0/1.0	0.12	CCNU (4 cycles)	12	SD	stable disease
4	1	0.6	0.3	2	0.5/0.2	0.06	ECT	13	CR	without recurrence
	2	1.2	0.8	2	0.5/0.4		Surgery		CR	without recurrence
5	1	2.9	0.6	2	0.5/0.5	0.03	Surgery	10	CR	without recurrence
6	1	0.03	0.005	4	0.1/0.1/0.1/0.1	0.07	/	44	SD	stable disease
	2	0.27	0.08	4	0.25/0.25/0.25/0.25		/		SD	
7	1	25.4	18.0	3	1.0/0.6/0.6	0.12	Surgery	2	PD	Euth. due to PD
8	1	0.45	0.6	1	0.2	0.025	CCNU (3 cycles)	5	PD	Euth. due to PD
	2	0.03	0.03	1	0.1					

Euth, euthanasia; MCT, mast cell tumor; CR, complete response; CCNU, lomustine; ECT, electrochemotherapy; SD, stable disease; PD, progressive disease

1 The smallest volume of tumor nodule, achieved 1 week after the last performed EGT session

2 Cumulative dose of received plasmid per kg of body weight

**Table 3.** Haematology and biochemistry profile of patient No. 3 with the highest number of serum samples, positive to IL-12 and IFN-γ

		**DAY 0**	**DAY 7**	**DAY 14**	**DAY 28**	**REFERENCE VALUES**
**Cytokine conc.**	IL-12 (pg/ml)	0	2.3	6.1	12.2	N/A
	IFN-γ (pg/ml)	0	388.1	179.6	164.3	N/A
**Haematology**	WBC (×10^9^/l)	4.75	6.14	5.98	6.3	6–17
	RBC (× 10^12^/l)	5.41	5.38	8.1	7.97	5.5–8.5
	HCT (L/L)	0.38	0.36	0.58	0.57	0.37–0.55
	PLT (× 10^9^/l)	287	283	303	224	200–500
**Biochemistry panel**	BUN (mmol/l)	3.28	4.2	3.94	N/D	2.5–9.6
	Crea (μmol/l)	74.54	75.7	71.23	N/D	44.2–132.6
	SAP (U/l)	47.3	27.5	23.8	47.1	20–156
	ALT (U/l)	21.8	64.0	58.6	217.6	21–102

WBC, white blood cells; RBC, red blood cells; HCT, haematocrit; PLT, platelets; BUN, blood urea nitrogen, Crea, creatinine; SAP, serum alkaline phosphatase; ALT, alanin aminotransferase; N/D, not detected
